# Chemical Space Exploration and Machine Learning-Based Screening of PDE7A Inhibitors

**DOI:** 10.3390/ph18040444

**Published:** 2025-03-21

**Authors:** Yuze Li, Zhe Wang, Shengyao Ma, Xiaowen Tang, Hanting Zhang

**Affiliations:** 1 Department of Pharmacology, School of Pharmacy, Qingdao University, Qingdao 266071, China; yuzeli136@163.com (Y.L.); wangzhe@qdu.edu.cn (Z.W.); cnsdwfmsy@163.com (S.M.); 2 Shandong Provincial Key Laboratory of Pathogenesis and Prevention of Brain Diseases, Qingdao University, Qingdao 266071, China; 3 Department of Medical Chemistry, School of Pharmacy, Qingdao University, Qingdao 266071, China

**Keywords:** phosphodiesterase 7A inhibitor, chemical informatics, machine learning, SHAP, virtual screening

## Abstract

**Background/Objectives**: Phosphodiesterase 7 (PDE7), a member of the PDE superfamily, selectively catalyzes the hydrolysis of cyclic adenosine 3′,5′-monophosphate (cAMP), thereby regulating the intracellular levels of this second messenger and influencing various physiological functions and processes. There are two subtypes of PDE7, PDE7A and PDE7B, which are encoded by distinct genes. PDE7 inhibitors have been shown to exert therapeutic effects on neurological and respiratory diseases. However, FDA-approved drugs based on the PDE7A inhibitor are still absent, highlighting the need for novel compounds to advance PDE7A inhibitor development. **Methods**: To address this urgent and important issue, we conducted a comprehensive cheminformatics analysis of compounds with potential for PDE7A inhibition using a curated database to elucidate the chemical characteristics of the highly active PDE7A inhibitors. The specific substructures that significantly enhance the activity of PDE7A inhibitors, including benzenesulfonamido, acylamino, and phenoxyl, were identified by an interpretable machine learning analysis. Subsequently, a machine learning model employing the Random Forest–Morgan pattern was constructed for the qualitative and quantitative prediction of PDE7A inhibitors. **Results**: As a result, six compounds with potential PDE7A inhibitory activity were screened out from the SPECS compound library. These identified compounds exhibited favorable molecular properties and potent binding affinities with the target protein, holding promise as candidates for further exploration in the development of potent PDE7A inhibitors. **Conclusions**: The results of the present study would advance the exploration of innovative PDE7A inhibitors and provide valuable insights for future endeavors in the discovery of novel PDE inhibitors.

## 1. Introduction

Phosphodiesterases (PDEs) constitute a superfamily of enzymes intricately involved in the regulation of various physiological functions and processes, such as central nervous system (CNS) functions, cancer development, and inflammation [1,2,3,4]. PDEs achieve this regulatory role by specifically hydrolyzing the second messengers, cyclic adenosine 3′,5′-monophosphate (cAMP) and cyclic guanosine 3′,5′-monophosphate (cGMP) [5,6]. In addition, the proteins associated with the cAMP and cGMP signaling pathways, such as the cAMP response element-binding protein (CREB), serve as critical targets in various diseases [7]. The PDE superfamily, comprising 11 subfamilies classified primarily based on their structures, substrates, and distribution, exhibits diverse substrate specificities [4,6]. Notably, PDE1, -2, -3, -10, and -11 hydrolyze both cAMP and cGMP indiscriminately, whereas PDE4, -7, and -8 are cAMP-specific, while PDE5, -6, and -9 are cGMP-specific hydrolases [2]. PDEs have emerged as therapeutic targets for a variety of diseases, with inhibitors showcasing their efficacy across various conditions [4,5,6]. For example, PDE1 inhibitors have been shown to mitigate adipogenesis in mice by modulating lipolysis and adipogenic cell signaling [8]. PDE5 inhibitors are effective in treating both erectile dysfunction and heart failure [9,10]. PDE8 inhibitors have been demonstrated to be effective in addressing vascular dementia [11], and PDE10 inhibitors exhibit the potential to ameliorate the symptoms associated with Huntington’s disease [12]. Given the wide-ranging therapeutic implications of these findings, PDEs have been recognized as pivotal targets for advancements in medicinal developments.

Among the PDE subtypes, PDE4 and PDE7 are prominently expressed in the brain and play pivotal roles in the diverse physiological processes associated with CNS functions. In addition, the concentration of cAMP also plays a significant regulatory role in bone formation processes [13]. These enzymes constitute critical targets for addressing CNS disorders [14,15]. Previous investigations have highlighted the potential efficacy of PDE4 inhibitors in treating Alzheimer’s disease, enhancing cognitive function, and managing alcohol addiction [15]. However, the clinical utility of PDE4 inhibitors has been challenged by the adverse effects, like emesis and vomiting [15]. Another cAMP-specific PDE subtype, PDE7, is emerging as a novel target in neuropharmacological drug development. The two subtypes of PDE7, PDE7A and PDE7B, which are encoded by the distinct genes, are expressed widely in the CNS but distributed variously in different brain regions. Specifically, PDE7A is broadly distributed in the cerebral cortex, hypothalamus, hippocampus, striatum, and thalamus, which makes PDE7A more prominent in the study of CNS disorders [14]. Similar to PDE4, PDE7 has also garnered significant research interest for its regulatory role in the CNS [15]. In addition, experimental findings suggest that inhibiting PDE7A can effectively protect the SH-SY5Y cell injury model induced by 6-OHDA without compromising the anesthesia duration induced by ketamine and xylazine, indicating the potential of PDE7A inhibitors in treating neurological diseases with fewer undesirable reactions, such as emesis [16].

**Figure 1:**
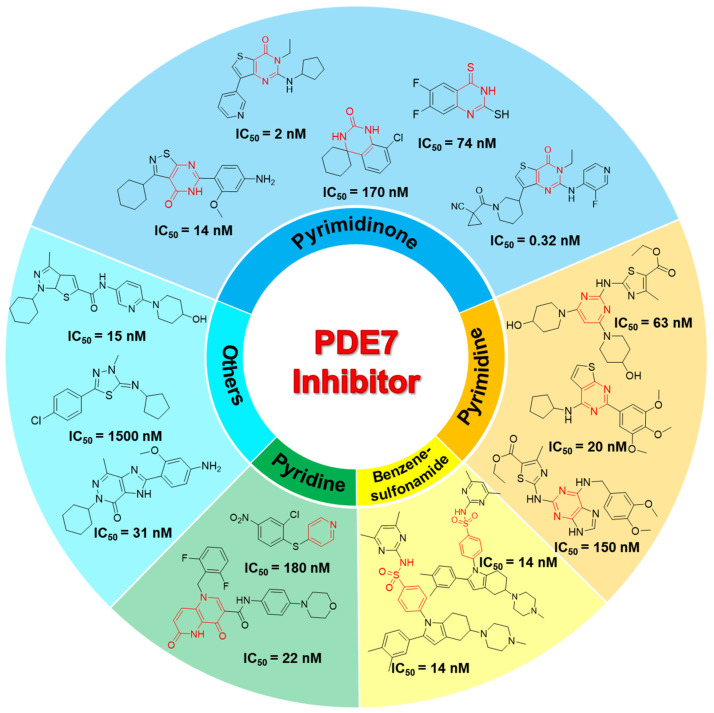
Exhibition and classification of the PDE7A inhibitors. Characteristic skeletons are labeled in red.

Since the identification of the first PDE7 inhibitor in 2000, diverse methodological approaches have been employed in PDE7 inhibitor development [17,18]. For example, ligand-based screening methods led to the discovery of potent PDE7A inhibitors, including thiadiazine and quinazoline derivatives, with notable anti-inflammatory effects in murine models [17]. Integrating pharmacophore screening and chemical modification yielded 3,4,5-trimethoxybenzyl 5-phenyl-2-furoate, a furan derivative with an IC_50_ of 5.17 μM against PDE7A, showing therapeutic efficacy in a murine model of multiple sclerosis [18]. Up to now, numerous compounds exhibiting inhibitory activity against PDE7A have been documented, falling into diverse categories, including pyrimidinones [19] and quinazolinones [20], pyrimidine compounds [21], pyridines [22], pyridinone analogs [16], and benzenesulfonamide derivatives [23] (Figure 1). Despite the inspiring advancements, FDA-approved drugs based on PDE7 inhibitors are still absent, highlighting the need for new screening techniques and novel compounds to advance PDE7A inhibitor development for associated diseases.

In contrast to traditional high-throughput screening, computer-aided drug design (CADD) has demonstrated rapid and efficient characteristics, becoming a vital approach in drug development [24]. Cheminformatics analysis and artificial intelligence-based drug design (AIDD) methods have recently rejuvenated drug development [25,26]. This current study analyzed the chemical information of the PDE7A inhibitors, establishing a correspondence between scaffolds, fragments/substructures, and PDE7A inhibitory activity. Subsequently, a machine learning-based predictive model for the PDE7A inhibitors was constructed, leading to the screening of novel compounds with potential PDE7A inhibitory activity. This study demonstrated significant potential for advancing the development of PDE7 inhibitors and their related therapeutic applications.

## 2. Results and Discussion

### 2.1. Chemical Information Analysis

**Figure 2:**
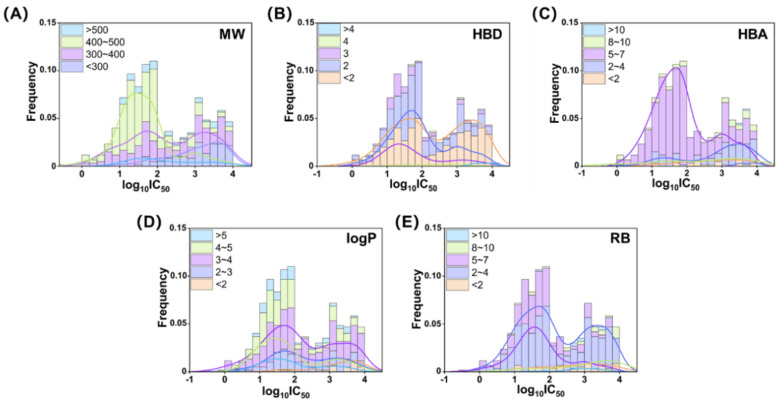
Distributions of PDE7A inhibitory activity against molecular weight (**A**), the number of hydrogen bond donors (**B**) and acceptors (**C**), logP (**D**), and the number of rotatable bonds (**E**). The activity was presented in logarithmic form (log_10_ IC_50_), and the unit was used in nM.

In the present study, we initiated a chemical property analysis based on Lipinski’s Rule of Five for the 596 active compounds in the database. As illustrated in Figure 2, the majority of active compounds comply with the rules, demonstrating favorable drug-likeness for PDE7A inhibitors. Specifically, these compounds exhibited characteristics such as a molecular weight (MW) of less than 500, a number of hydrogen bond donors (HBDs) of less than five, a number of hydrogen bond acceptors (HBAs) of less than 10, a water partition coefficient (logP) of less than five, and a number of rotatable bonds (RB) of less than 10.

Further exploration revealed that compounds with a MW between 400 and 500 generally displayed higher inhibitory activity against PDE7A (IC_50_, 10~100 nM). As depicted in Figure 2A, the inhibitory activity exhibited a decreasing trend as the MW decreased, with compounds having a MW below 300 showing very low or even no activity (IC_50_, 1~10 μM). In Figure 2B, the distribution of HBDs for the PDE7A active compounds primarily centered around three or below. Notably, the compounds with an HBD frequency of two demonstrated high inhibitory activity, while those with an HBD frequency of less than two exhibited a bimodal distribution, indicating no clear preference for PDE7A inhibitory activity in this subset. Additionally, the compounds with an HBD frequency of three displayed a relatively small but significantly high activity, suggesting a potential source of potent PDE7A inhibitors. The distribution of HBAs in the 596 active compounds revealed concentrations of between two and seven. Higher PDE7A inhibitory activity was observed in the range of 5~7, while lower activity was observed below 4. Figure 2D illustrated a positive correlation between logP and PDE7A inhibitory activity, emphasizing the importance of hydrophobicity for PDE7A inhibitors. Compounds with a logP in the range of 4~5 were concentrated in the high-activity area. As the logP decreased, the distribution curve shifted to the right, and compounds with a logP of less than two were nearly inactive. An analysis of the rotatable bonds (RB) in Figure 2E showed a concentration in the range of 2~7. Within this range, compounds with greater molecular flexibility (higher RB, 5~7) were more likely to exhibit higher PDE7A inhibitory activity.

In summary, the compounds displaying high PDE7A inhibitory activity (IC_50_ < 100 nM) in the database generally possessed characteristics, such as higher molecular weight (MW, 400~500) and hydrophobicity (logP, 4~5), a moderate number of hydrogen bond donors (HBDs, 2~3) and acceptors (HBAs, 5~7), and moderate molecular flexibility (RB, 5~7). These findings provide valuable insights into the structural and physicochemical attributes associated with potent PDE7A inhibition.

### 2.2. Murcko Scaffold Analysis

**Figure 3:**
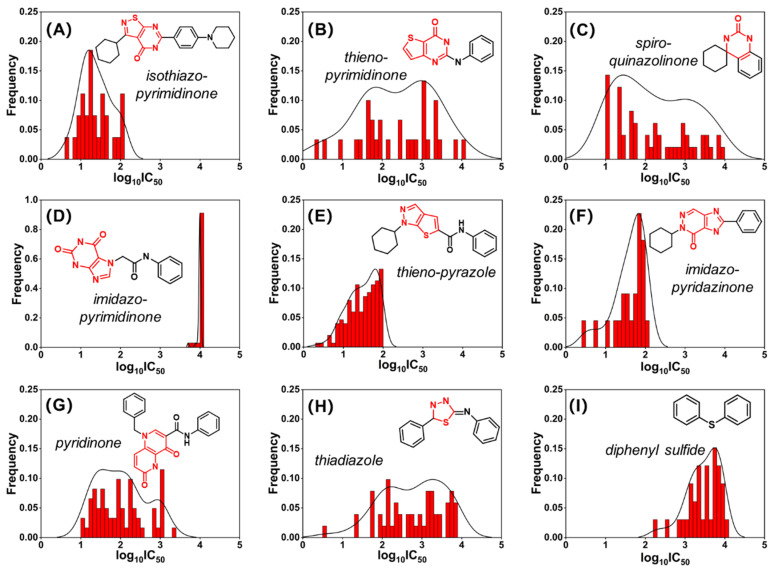
The most prevalent Murcko scaffolds among the active compounds in the database. The structure and name of scaffold are displayed in every subfigure. The scaffold feature was labeled in red. The red bars and black lines represent the frequency of the compounds at different activity values. The activity was presented in logarithmic form (log_10_ IC_50_), and the unit was used in nM.

Nine of the most prevalent Murcko scaffolds among the 752 compounds in the database (596 active and 156 inactive) are illustrated in Figure 3. Eight of these scaffolds (Figure 3A–H) were nitrogen-containing heterocyclic structures, such as pyrimidinone and pyridinone, aligning with previously reported PDE7A inhibitors summarized in Figure 1. Notably, diphenyl sulfide compounds, a rarity in previous studies, were discovered to exhibit slight PDE7A inhibitory activity at the micromolar scale (Figure 3I).

Among the four pyrimidinone scaffolds, isothiazo-pyrimidinone stood out as a prominent scaffold, with the compounds containing it displaying high inhibitory activity toward PDE7A (IC_50_ < 100 nM), as shown in Figure 3A. Conversely, the compounds containing the imidazo-pyrimidinone scaffold exhibited extremely weak or even no PDE7A inhibitory activity (IC_50_ ~10 μM), consistent with the relatively weak activity reported for IBMX (8.10 μM) [27] and guanine-based inhibitors (4.88 μM) [28] in previous investigations. Additionally, two other scaffolds, thieno-pyrazole and imidazo-pyridazinone, also demonstrated remarkably high PDE7A inhibitory activity similar to isothiazo-pyrimidinone. Intriguingly, all three high-activity scaffolds (isothiazo-pyrimidinone, thieno-pyrazole, and imidazo-pyridazinone) shared a cyclohexyl-nitrogenous heterocyclic ring structure. This structural motif could potentially serve as a characteristic fragment for the further evaluation, screening, and design of PDE7A inhibitors. These findings provide valuable insights into the diversity of the scaffolds contributing to PDE7A inhibitory activity, with potential implications for the identification of novel structures and structural motifs for future drug development targeting PDE7A.

### 2.3. Development and Characterization of Machine Learning Models

A substantial volume of PDE7A inhibition assay data sourced from ChEMBL and PubChem facilitated the construction of a machine learning-based predictive model for PDE7A inhibitors. The present study employed a concatenated pattern, integrating qualitative prediction through a classification model and quantitative prediction through a regression model, for the construction of the PDE7A inhibitors predictive model. The performance of these models was rigorously assessed through internal and external validation.

**Figure 4:**
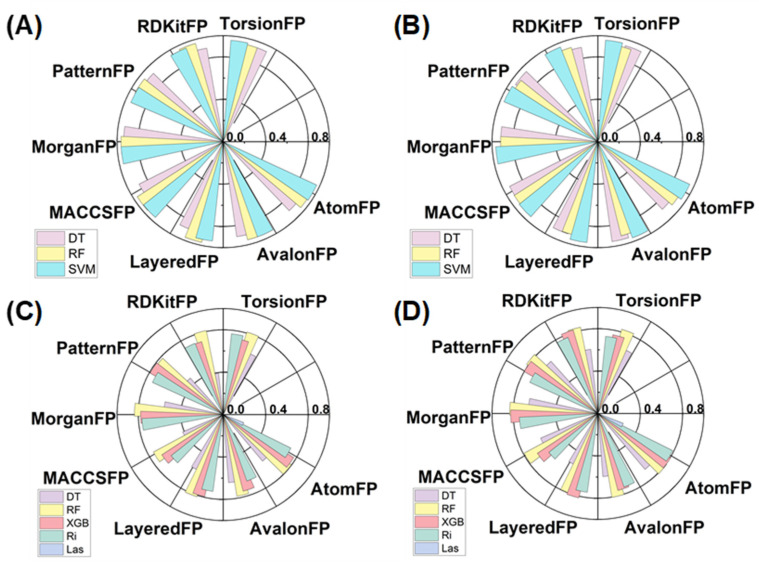
The performances of the machine learning models trained using different algorithms based on the different molecular fingerprints. The performances of the classification and regression models were evaluated with Accuracy and R^2^, respectively. Classification models in internal (**A**) and external (**B**) validation, regression models in internal (**C**) and external (**D**) validation.

Utilizing three classical algorithms and eight molecular fingerprints, a total of 24 machine learning-based classification models were developed for the qualitative prediction of PDE7A inhibitors. The results illustrated that all 24 models exhibited remarkably high accuracy levels during both internal validation (Figure 4A and Table S3, 0.88~0.97) and external validation (Figure 4B and Table S4, 0.88~0.95). Additional evaluation criteria, including precision, recall value, and F1 score, were also consistently high, as listed in Tables S3 and S4. The outstanding performance of these models indicated their qualification for the qualitative prediction of PDE7A inhibitors, demonstrating strong generalization capabilities.

Employing the same molecular-fingerprints scale, five machine learning algorithms were introduced for constructing regression models of the PDE7A inhibitors. As depicted in Figure 4C,D and Tables S5 and S6, R^2^ of the regression models constructed with the XGBoost, Random Forest and Ridge algorithms (mostly more than 0.70 in both internal and external validation) outperformed those with decision tree and Lasso algorithms (mostly less than 0.50 in both validations). Model stability assessments through RMSE and MAE also underscored the superiority of the XGBoost, Random Forest, and Ridge algorithms (Tables S5 and S6). Overall, the exceptional performance of the Random Forest and XGBoost algorithms highlighted the advantage of multiple decision trees in handling complex regression tasks. Notably, no significant disparity was observed in the models using different fingerprints, indicating that the choice of machine learning algorithm may outweigh the influence of fingerprints in constructing regression models of PDE7A inhibitors.

Considering the comprehensive performance, the model generated by the Random Forest algorithm paired with the Morgan fingerprint (RF–Morgan) exhibited notable accuracy (R^2^ is over 0.80, the highest in all the models of both internal and external validation) and high stability (shows the lowest RMSE and MAE level in the two validations). Consequently, the RF–Morgan model was selected as the quantitative model for predicting PDE7A inhibitors. Furthermore, all the constructed classification models demonstrated exceptional performance in qualitatively predicting PDE7A inhibitors. Accordingly, the selected RF–Morgan model was recommended for qualitative prediction in tandem with its role in quantitative prediction. Ultimately, the RF–Morgan model was employed in subsequent interpretable machine learning analyses and further integrated into the concatenated pattern (qualitative in series with quantitative) for the prediction and screening of PDE7A inhibitors.

### 2.4. Interpretable Machine Learning Analysis

**Figure 5:**
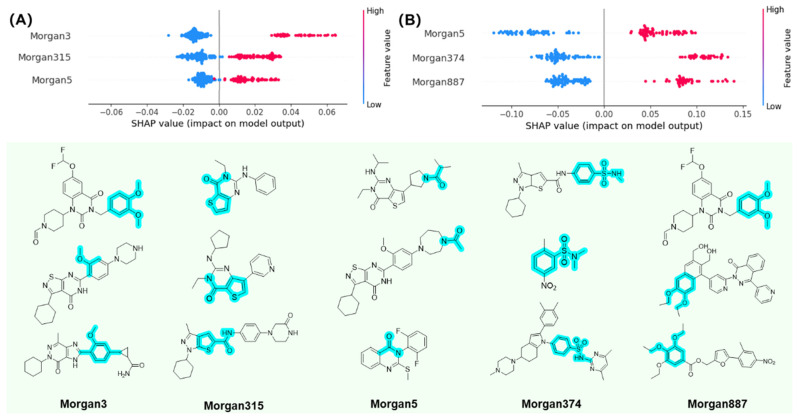
The significant active substructures calculated by SHAP in classification (**A**) and regression (**B**) model. The substructures indicated by SHAP were highlighted in cyan.

To enhance the interpretability of the PDE7A inhibitor prediction model, the SHapley Additive exPlanations (SHAP) method was introduced to elucidate the relationship between compound substructures and inhibitory activity on PDE7A. Figure 5 illustrates the top three substructures with positive correlations on PDE7A inhibitory activity as calculated by SHAP in both the classification and regression models.

In the SHAP analysis of the classification model (Figure 5A), Morgan3, referring to the phenoxyl substructure, displayed a significantly high SHAP value, emphasizing the phenoxyl group as an important feature in PDE7A inhibitors. Meanwhile, Morgan315 and Morgan5, representing thiophenecarboxamide and acylamino substructure, also exhibited high SHAP values, suggesting that the acylamino group tends to be another promoter toward PDE7A inhibition. For the regression model displayed in Figure 5B, Morgan5 was identified with a significant influence on PDE7A inhibitory activity once again. Actually, acylamino is the most common substructure in PDE7A inhibitors. For example, two types of conventional PDE7A inhibitors, pyrimidinone and pyridinone compounds, exactly hold acylamino as their characteristic group [16,19]. As another substructure indicated by SHAP analysis, Morgan374, representing the benzenesulfonamido groups, is also commonly found in PDE7A inhibitors. For instance, BRL-50481, a widely used PDE7A inhibitor [23], is a typical benzenesulfonamide compound. Additionally, Morgan887, which shares structural similarities with Morgan3, also exhibited considerable impact on PDE7A inhibitory activity, which highlights the inhibitory effect of phenoxyl-based substructures on PDE7A. It is noteworthy that phenoxyl compounds have been infrequently reported in PDE7A inhibitor-related research [29], suggesting the potential to provide new insights for the design and development of future PDE7A inhibitors. The interpretability offered by the SHAP analysis enhances the understanding of the key substructures influencing PDE7A inhibitory activity, facilitating more informed decisions on the design and optimization of the compounds targeting PDE7A.

### 2.5. PDE7A Inhibitor Screening

**Figure 6:**
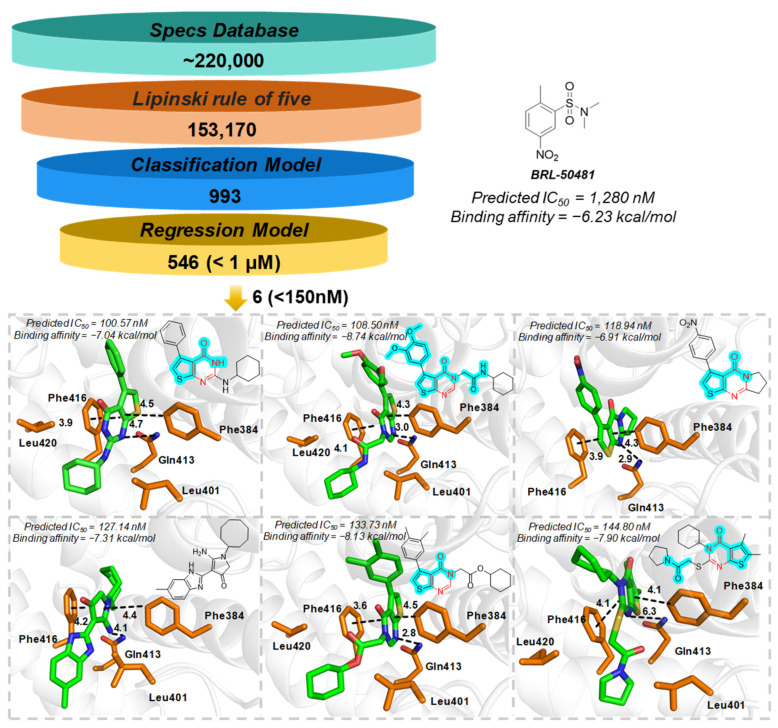
The crateriform screening pattern for the PDE7A inhibitors and six identified compounds. The positive control (BRL-50481) was present in right. The pyrimidinone scaffold was labeled in red and the significant substructures were highlighted in cyan. The ligand-receptor interactions were shown with stick model, in which the carbon atom was colored in green and orange for compounds and the key residues respectively. Other atoms were uniformly colored (red for oxygen, blue for nitrogen and yellow for sulphur atom). The interactions were labeled in black dotted line and the unit of distance was used in Å.

Utilizing the explored chemical spaces and the constructed predictive model for PDE7A inhibitors, a crateriform screening pattern, incorporating Lipinski’s Rule of Five, machine learning-based qualitative and quantitative prediction was employed for screening the SPECS commercial compound library, which consists of approximately 220,000 compounds. As displayed in Figure 6, the SPECS compound library underwent an initial filtration based on Lipinski’s Rule of Five, resulting in ~150,000 retained compounds. Subsequently, the RF–Morgan-based qualitative prediction in series with quantitative prediction was utilized to predict the inhibitory activity and specific IC_50_ values on PDE7A. This process yielded 546 compounds with predicted IC_50_ values below 1 μM.

In light of the statistical challenges presented by the abundance of compounds generated from the regression model and the pursuit of identifying more potent PDE7A inhibitors, a refined screening criterion of 150 nM was adopted, leading to the identification of six compounds. As depicted in Figure 6, five out of the six identified compounds were categorized as pyrimidinone derivatives, a series of compounds previously documented for their inhibitory activity against PDE7A. This observation underscores the robust generalization capability of our machine learning model in efficiently screening for PDE7A inhibitors. Additionally, compound four may offer novel scaffolds for the exploration of PDE7A inhibitors. Of particular note is that the six identified compounds encompass substructures identified as crucial contributors to PDE7A inhibitory activity, such as acylamino and phenoxyl, as highlighted in Figure 6. This implies once again the potential inhibitory activity against PDE7A, and the molecular properties of the six compounds approximately adhere to the refined Lipinski’s Rule of Five criteria, indicating both high PDE7A inhibitory activity and potential druggability.

Further ligand-protein interactions revealed that Phe384 and Phe416 clamp the aromatic skeleton of ligand through π–π interactions with a sandwich pattern, and Gln413 provide polar interactions with the pyrimidinone scaffold. Some aliphatic residues, like Leu401 and Leu420, form hydrophobic interactions with the aliphatic groups. The ligand-protein interactions above are consistent with the previously reported interactions of pyrimidinones with PDE7A [27,30,31]. Compared to the commonly employed PDE7A inhibitor BRL-50481, all six compounds exhibited higher performances of both the binding affinity (docking score: about −7.00 kcal/mol vs. −6.31 kcal/mol) and predicted inhibitory activity (IC_50_: about 100 nM vs. 1280 nM) toward the target protein. This suggests the promising potential of the six compounds to serve as more potent PDE7A inhibitors.

## 3. Materials and Methods

### 3.1. Data Preparation

**Figure 7:**
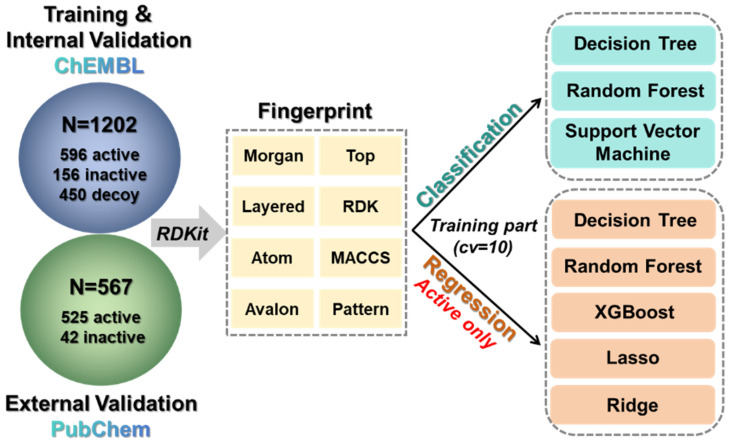
Composition of the database and the workflow of machine learning.

The data utilized here were sourced from the PDE7A inhibition assay data available in ChEMBL and PubChem [32,33], two open-source database websites that have been widely used with strong applicability [34,35,36]. ChEMBL played a primary role in cheminformatics analysis and in the construction of both the training and internal validation sets for machine learning, while PubChem contributed to the formation of the machine learning external validation set, supplemented by additional chemical structures obtained from previous literature [37] (detailed data were listed in the Supplementary Materials_S1). Before embarking on data analysis, a thorough database curation process was initiated to ensure data quality. This process involved the following steps (1) Retention of only compound structural information (SMILES) and activity information (IC_50_), with the presentation of activity values in logarithmic form (−log_10_ IC_50_) [38,39]. (2) Elimination of compounds with incomplete information, such as those lacking structural information or containing non-numeric data. In cases of compounds with repetitive information, if the activity values differed by less than ten-fold, the average value was computed; otherwise, the compound was excluded. (3) Establishment of an activity threshold of 10 μM of IC_50_, wherein compounds with values below 10 μM were categorized as active, while those equaled or greater than 10 μM were labeled as inactive [40]. In addition, 450 decoys obtained from DUD-E (A Database of Useful Decoys: Enhanced, https://dude.docking.org/ (accessed on 2 October 2024)) [41] were introduced, due to the imbalanced number of active and inactive data. Ultimately, a dataset with 1202 compounds (596 active and 156 inactive compounds as well as 450 decoys) was collected for the further training and testing (internal validation) of the machine learning model (Figure 7). The dataset was partitioned into training and testing sets in an 80% to 20% ratio, with the training phase further utilizing 10-fold cross-validation. Additionally, another dataset consisting of 567 compounds, comprising 525 active and 42 inactive compounds, was designated to evaluate the performance of the machine learning model (external validation, Figure 7). In addition, the active compounds extracted from the training, testing and validation sets were employed for cheminformatics analysis.

### 3.2. Molecular Features and Fingerprint Calculation

In the present study, Lipinski’s Rule of Five and the Murcko scaffold were utilized to characterize the molecular features in the database [42,43]. During the extraction of the Murcko scaffolds, the scaffolds with a similarity greater than 0.5 were merged. To represent the molecular structures, eight molecular fingerprints were selected, namely RDKitFP, MorganFP, AtomFP, AvalonFP, MACCSFP, PatternFP, LayeredFP, and TorsionFP. During the generation of the molecular fingerprints for the compounds in the database, the missing values were input with 0, and variance filtering was applied in cases where the variance was 0 [44]. Additional information on the molecular fingerprints is shown in Table S1. In addition, an RDKit was employed for the generation of all the molecular features and fingerprints [34].

### 3.3. Machine Learning Model Construction

As depicted in Figure 7, a combined qualitative (classification model) and quantitative (regression model) scheme based on machine learning was implemented for the construction of a PDE7A inhibitor prediction model. Three algorithms (Decision Tree [45], Random Forest [46], and Support Vector Machine [47]) were used for the classification model, while five algorithms (Decision Tree, Random Forest, XGBoost [48], Lasso [49], and Ridge regression [50]) were employed for the regression model. Based on eight different molecular fingerprints, we generated a total of 24 classification models and 40 regression models. Each of these models was thoroughly evaluated to establish the final PDE7A inhibitor prediction and screening scheme. The development of machine learning models utilized Scikit-learn on the Python platform (version 3.11) [51], employing a greedy strategy for optimizing hyperparameters. The best hyperparameter values for the optimal machine learning model were listed in Table S2 and that for other constructed models were summarized in the Supplementary Materials_S2.

### 3.4. Model Evaluation

The predictive ability and robustness of the constructed models were assessed through 10-fold cross-validation (internal validation) and external validation. Four evaluation criteria, referred to as Precision, Recall, Accuracy, and F1 Score were introduced to evaluate the performance of the classification model. The calculation methods of them are listed as follows [52]:

(1) Precision=TP/(TP+FP)

(2) Recall=TP/(TP+FN)

(3) Accuracy=(TN+TP)/(TN+TP+FN+FP)

(4) F1 Score=(2×Precision×Recall)/(Precision+Recall)

where, TP (true positive) represents correctly predicted positive data, FP (false positive) denotes positive data with incorrect predictions, TN (true negative) signifies correctly predicted negative data, and FN (false negative) indicates negative data with incorrect predictions. Generally, higher Accuracy and F1 Score values tend to reflect a stronger generalization ability of the model [52].

The other three evaluation criteria that refer to the coefficient of determination (R^2^), root mean square error (RMSE), and mean absolute error (MAE) were used to assess the performance of the regression model. The calculation methods of them are listed as follows [52]:

(5) $$R²=1-\frac{\sum_{i}(y_{i}-\hat{y_{i}})²}{\sum_{i}(y_{i}-\bar{y_{i}})²}$$

(6) $$RMSE=\sqrt{\frac{1}{m}\sum_{i=1}^{m}(y_{i}-\bar{y_{i}})²}$$

(7) $$MAE=\frac{1}{m}\sum_{i=1}^{m}\left|y_{i}-\hat{y_{i}}\right|$$

where, *m* represents the total number of samples, $y_{i}$, $\bar{y_{i}}$ and $\hat{y_{i}}$ are the true value, average value, and predicted biological activity value of the molecule, respectively. In general, a higher R^2^ (closer to 1) indicates greater precision, while a smaller RMSE or MAE (closer to 0) suggests better stability [52].

### 3.5. Feature Importance Analysis

The SHapley Additive exPlanations (SHAP) method was employed to enhance the interpretability of the machine learning model. SHAP values, derived from coalitional game theory, were used to analyze the contribution of each feature to the model output for various combinations of feature values. The calculation involves combining and weighting model outputs for different feature value combinations to determine the marginal contribution of each feature value to the output. This analysis aids in understanding the influence of each feature in a specific prediction and explaining the overall prediction process of the model. SHAP calculates the importance of the features by a model [53]:

(8) $$f\left(x\right)\approx g\left(z^{\prime }\right)=\emptyset_{0}+\sum_{i=1}^{M}\emptyset_{i}z_{i}^{\prime }$$

where, ∅_0_ represents the average value of all sample target variables, $\emptyset_{i}$ is the SHAP value of descriptor i, which represents the contribution of descriptor i to the final prediction result, $z_{i}^{\prime }$ ∈ {0, 1} indicates the presence (1) or absence (0) of descriptor i, *M* is the total number of molecular descriptors and $f\left(x\right)$ is the output of the original model, SHAP makes the sum of all descriptors $g\left(z^{\prime }\right)$ attributions approximately equal to it [53].

### 3.6. Molecule Docking

The initial receptor model was constructed based on the PDE7A crystal structure (PDB ID: 4Y2B) [30]. Protein and ligand parts were extracted and processed for subsequent molecular docking experiments. The compounds screened by the machine learning model underwent further optimization using the OPLS4 force field. Protonated states of ionizable groups were defined at pH 7.0 ± 0.2, which simulated the slightly fluctuating pH conditions in the physiological environment. The protonated states of titratable residues in the receptor structure were also calculated at the same pH for ligand preparation. The molecular docking analysis utilized AutoDock Vina v1.5.7 [54], where the centroid of the ligand was defined as the center of the docking grid and the size of the grid was set to 25 × 25 × 25 Å^3^. Finally, the flexible molecular docking based on the induced fit theory was executed, and the results (binding mode and docking score) with the best docking score were recorded.

## 4. Conclusions

PDE7 inhibitors represent a novel class of drugs with promising therapeutic potential, particularly in the treatment of CNS disorders. In the present study, we conducted a cheminformatics analysis to elucidate the chemical characteristics of highly active PDE7A inhibitors. We also identified the scaffolds prevalent in existing PDE7A inhibitors and further recognized isothiazo-pyrimidinone, thieno-pyrazole and imidazo-pyridazinone derivatives as highly PDE7A inhibitory compounds. Additionally, our analysis identified specific substructures that potentially enhance the activity of PDE7A inhibitors. Subsequently, employing machine learning methods, robust RF–Morgan-based classification and regression models showcasing strong generalization capabilities, were successfully established. Utilizing these models, a refined screening of the SPECS compound library was established and used to identify six compounds with potential PDE7A inhibitory activity. These compounds, exhibiting favorable molecular properties and enhanced binding affinity in comparison to known PDE7 inhibitors, hold a promise as candidates for further exploration in the development of potent PDE7A inhibitors. However, it should be noted that the predictive capability of our current model is specifically limited to PDE7A inhibitors, as it lacks generalizability across other PDE subtypes. Furthermore, the practical application of this model necessitates experimental validation to confirm its reliability and accuracy. In summary, the present work not only statistically analyzed the existing PDE7A inhibitors, but also provided valuable insights and methodologies for future endeavors in the field of PDE inhibition and drug development.

## Data Availability

Data are provided within the manuscript and Supplementary Materials files.

## Supplementary Materials

The following supporting information can be downloaded at https://www.mdpi.com/article/10.3390/ph18040444/s1, detailed information on molecular fingerprints (Table S1), best hyperparameter of RF–Morgan in the classification and regression model (Table S2), performance of classification models in internal validation (Table S3) and external validation (Table S4), performance of regression models in internal validation (Table S5) and external validation (Table S6), list of training and validation set (Supplementary Materials_S1), and detailed hyperparameter of the machine learning models (Supplementary Materials_S2).
