# Supplementary material for: Chemical Space Exploration and Machine Learning-Based Screening of PDE7A Inhibitors
Source: Pharmaceuticals (Basel). 2025 Mar 21;18(4):444. doi: 10.3390/ph18040444 (PMC12030294; doi:10.3390/ph18040444)
Supplement: Supplementary file 1 [file pharmaceuticals-18-00444-s001.zip › Supporting Information-Tables S1-S6.pdf]

Supporting Information

Chemical Space Exploration and Machine Learning-Based Screening of PDE7A Inhibitors

Yuze Li[1], Zhe Wang[1,2], Shengyao Ma[1], Xiaowen Tang[2,3,]* and Hanting Zhang[1,2,]*

[1] *Department of Pharmacology, School of Pharmacy, Qingdao University, Qingdao 266071, China*

[2] *Shandong Provincial Key Laboratory of Pathogenesis and Prevention of Brain Diseases, Qingdao University, Qingdao 266071, China*

[3] *Department of Medical Chemistry, School of Pharmacy, Qingdao University, Qingdao 266071, China*

* To whom correspondence should be addressed:

Xiaowen Tang (xwtang1219@qdu.edu.cn); Hanting Zhang (htzhang@qdu.edu.cn)

Table of contents

Table S1……………………………………………………………………………S2

Table S2……………………………………………………………………………S3

Table S3……………………………………………………………………………S4

Table S4……………………………………………………………………………S5

Table S5……………………………………………………………………………S6

Table S6……………………………………………………………………………S7

Table S1. The full name, bits, and source of the fingerprints.

Abbreviation	Full Name	Bits	Source
MorganFP	Extended Connectivity Fingerprints	1024	https://rdkit.org/docs/index.html
RDKitFP	Topological Fingerprints	2048	https://rdkit.org/docs/index.html
AtomFP	Atom-Pair fingerprints	2048	https://rdkit.org/docs/index.html
TorsionFP	Topological Torsion fingerprints	2048	https://rdkit.org/docs/index.html
MACCSFP	Molecular Accession System Keys fingerprints	166	https://doi.org/10.1021/ci010132r
AvalonFP	Avalon fingerprints	2048	https://doi.org/10.1021/ci050413p
LayeredFP	Layered Fingerprints	2048	https://rdkit.org/docs/index.html
PatternFP	Pattern Fingerprints	2048	https://rdkit.org/docs/index.html

Table S2. Best hyperparameter of classification model and regression model.

Model	Algorithm-Fingerprint	Hyperparameter
Classification	Random Forest-Morgan	n_estimators=129<br>max_depth=17<br>max_features=48<br>min_samples_leaf=1
Regression	Random Forest-Morgan	min_samples_leaf=1<br>min_samples_split=2<br>max_depth=44<br>n_estimators=99<br>max_features=87

Table S3. Performance for classification models in internal validation.

Algorithm	Fingerprint	Precision	Recall	F1 Score	Accuracy
Decision Tree (DT)	Top	0.944	0.942	0.942	0.942
	RDK	0.896	0.896	0.896	0.896
	Pattern	0.922	0.921	0.921	0.921
	Morgan	0.938	0.938	0.938	0.938
	MACCS	0.891	0.888	0.888	0.888
	Layered	0.885	0.884	0.884	0.884
	Avalon	0.906	0.905	0.905	0.905
	Atom	0.905	0.900	0.900	0.900
Random Forest (RF)	Top	0.943	0.942	0.942	0.942
	RDK	0.963	0.963	0.963	0.963
	Pattern	0.950	0.950	0.950	0.950
	Morgan	0.963	0.963	0.963	0.963
	MACCS	0.963	0.963	0.963	0.963
	Layered	0.971	0.971	0.971	0.971
	Avalon	0.955	0.954	0.954	0.954
	Atom	0.959	0.959	0.958	0.959
Support Vector Machine (SVM)	Top	0.959	0.959	0.958	0.959
	RDK	0.951	0.950	0.950	0.950
	Pattern	0.948	0.946	0.946	0.946
	Morgan	0.964	0.963	0.963	0.963
	MACCS	0.959	0.959	0.958	0.959
	Layered	0.942	0.942	0.942	0.942
	Avalon	0.954	0.954	0.954	0.954
	Atom	0.971	0.971	0.971	0.971

Table S4. Performance for classification models in external validation.

Algorithm	Fingerprint	Precision	Recall	F1 Score	Accuracy
Decision Tree (DT)	Top	0.967	0.942	0.949	0.942
	RDK	0.930	0.903	0.914	0.903
	Pattern	0.955	0.947	0.950	0.947
	Morgan	0.959	0.919	0.931	0.919
	MACCS	0.956	0.935	0.942	0.935
	Layered	0.946	0.914	0.925	0.914
	Avalon	0.950	0.951	0.950	0.951
	Atom	0.944	0.882	0.902	0.882
Random Forest (RF)	Top	0.957	0.924	0.934	0.924
	RDK	0.961	0.924	0.935	0.924
	Pattern	0.960	0.929	0.939	0.929
	Morgan	0.961	0.919	0.931	0.919
	MACCS	0.958	0.940	0.946	0.940
	Layered	0.951	0.910	0.923	0.910
	Avalon	0.959	0.915	0.928	0.915
	Atom	0.960	0.921	0.932	0.921
Support Vector Machine (SVM)	Top	0.956	0.914	0.927	0.914
	RDK	0.953	0.915	0.927	0.915
	Pattern	0.962	0.942	0.948	0.942
	Morgan	0.943	0.907	0.919	0.907
	MACCS	0.949	0.942	0.945	0.942
	Layered	0.936	0.922	0.928	0.922
	Avalon	0.956	0.921	0.932	0.921
	Atom	0.949	0.938	0.943	0.938

Table S5. Performance for regression models in internal validation

Algorithm	Fingerprint	R^2	MAE	RMSE
Decision Tree (DT)	Top	0.627	0.444	0.586
	RDK	0.393	0.536	0.748
	Pattern	0.464	0.510	0.703
	Morgan	0.561	0.476	0.636
	MACCS	0.416	0.534	0.734
	Layered	0.575	0.498	0.626
	Avalon	0.644	0.454	0.573
	Atom	0.575	0.480	0.626
Random Forest (RF)	Top	0.810	0.360	0.441
	RDK	0.809	0.346	0.449
	Pattern	0.780	0.385	0.489
	Morgan	0.837	0.334	0.424
	MACCS	0.747	0.403	0.535
	Layered	0.814	0.342	0.437
	Avalon	0.782	0.362	0.473
	Atom	0.799	0.372	0.461
XGBoost (XGB)	Top	0.727	0.414	0.544
	RDK	0.717	0.395	0.547
	Pattern	0.793	0.343	0.462
	Morgan	0.778	0.388	0.478
	MACCS	0.692	0.448	0.577
	Layered	0.794	0.359	0.460
	Avalon	0.747	0.390	0.512
	Atom	0.778	0.356	0.474
Ridge (Ri)	Top	0.766	0.391	0.508
	RDK	0.723	0.336	0.461
	Pattern	0.733	0.380	0.512
	Morgan	0.767	0.390	0.507
	MACCS	0.626	0.503	0.636
	Layered	0.731	0.349	0.520
	Avalon	0.665	0.436	0.572
	Atom	0.712	0.427	0.544
Lasso (Las)	Top	0*	0.917	1.055
	RDK	0*	0.738	0.882
	Pattern	0*	0.854	1.000
	Morgan	0*	0.917	1.055
	LMACCS	0*	0.839	1.001
	Layered	0*	0.849	1.004
	Avalon	0*	0.839	1.001
	Atom	0.208	0.740	0.902

* Values below 0 are displayed as 0.

Table S6. Performance for regression models in external validation

Algorithm	Fingerprint	R^2	MAE	RMSE
Decision Tree (DT)	Top	0.653	0.429	0.568
	RDK	0.609	0.451	0.603
	Pattern	0.663	0.407	0.560
	Morgan	0.659	0.398	0.563
	MACCS	0.589	0.446	0.618
	Layered	0.531	0.495	0.661
	Avalon	0.597	0.469	0.613
	Atom	0.691	0.405	0.537
Random Forest (RF)	Top	0.825	0.254	0.403
	RDK	0.831	0.246	0.396
	Pattern	0.818	0.264	0.412
	Morgan	0.834	0.242	0.392
	MACCS	0.792	0.296	0.440
	Layered	0.799	0.312	0.432
	Avalon	0.802	0.309	0.429
	Atom	0.808	0.318	0.423
XGBoost (XGB)	Top	0.756	0.349	0.477
	RDK	0.822	0.287	0.407
	Pattern	0.801	0.261	0.430
	Morgan	0.825	0.275	0.404
	MACCS	0.685	0.385	0.542
	Layered	0.817	0.298	0.413
	Avalon	0.750	0.339	0.482
	Atom	0.805	0.314	0.426
Ridge (Ri)	Top	0.731	0.356	0.500
	RDK	0.772	0.312	0.461
	Pattern	0.707	0.365	0.522
	Morgan	0.743	0.339	0.489
	MACCS	0.591	0.481	0.617
	Layered	0.750	0.322	0.482
	Avalon	0.737	0.355	0.495
	Atom	0.788	0.318	0.444
Lasso (Las)	Top	0*	0.816	0.968
	RDK	0*	0.827	0.972
	Pattern	0*	0.828	0.973
	Morgan	0*	0.816	0.968
	LMACCS	0*	0.823	0.971
	Layered	0*	0.823	0.971
	Avalon	0*	0.823	0.971
	Atom	0.258	0.671	0.831

* Values below 0 are displayed as 0.
